# Association of food access and neighbor relationships with diet and underweight among community-dwelling older Japanese

**DOI:** 10.1016/j.je.2016.12.016

**Published:** 2017-06-17

**Authors:** Hideko Nakamura, Mieko Nakamura, Eisaku Okada, Toshiyuki Ojima, Katsunori Kondo

**Affiliations:** a School of Nursing, Seirei Christopher University, Shizuoka, Japan; b Department of Community Health and Preventive Medicine, Hamamatsu University School of Medicine, Shizuoka, Japan; c Center for Preventive Medical Sciences, Chiba University, Chiba, Japan; d Department of Gerontological Evaluation, Center for Gerontology and Social Science, National Center for Geriatrics and Gerontology, Aichi, Japan

**Keywords:** Older people, Food access, Neighbors, Diet

## Abstract

**Background:**

Food access is important for maintaining dietary variety, which predicts underweight. The aim of this study was to examine the association of food access and neighbor relationships with eating and underweight.

**Methods:**

We analyzed cross-sectional data from 102,869 Japanese individuals aged 65 years or older. The perceived availability of food was assessed using the presence or absence of food stores within 1 km of the home. Level of relationships with neighbors was also assessed. The odds ratios (ORs) and 95% confidence intervals (CIs) for infrequent food intake and underweight were determined using logistic regression analysis.

**Results:**

The proportion of men and women having low access to food was 25–30%. Having low food access (OR 1.18; 95% CI, 1.12–1.25 for men and OR 1.26; 95% CI, 1.19–1.33 for women) and a low level of relationship with neighbors (OR 1.38; 95% CI, 1.31–1.45 for men and OR 1.57; 95% CI, 1.48–1.67 for women) was associated with infrequent intake of fruits and vegetables in both sexes. Association between low food access and infrequent intake of fruits and vegetables was higher among men with low levels of neighbor relationship (OR 1.34; 95% CI, 1.23–1.46) than among men with high levels of relationship (OR 1.10; 95% CI, 1.03–1.18).

**Conclusions:**

Low perceived availability of food is a risk factor for low dietary variety among older people. Furthermore, high levels of relationship with neighbors may relieve the harmful effect of low food access.

## Highlights

• Low food access was associated with infrequent eating of foods in older Japanese.
• Low level of relationship with neighbors was also associated with infrequent eating.
• Interaction between food access and neighbor relationships was significant in men.

## Introduction

In a super-aged society, such as Japan, the extension of healthy life expectancy is one of the most important objectives for health care.^1^ For older people, low dietary variety is related to functional status decline^2^, ^3^, ^4^, ^5^ and predicts underweight,^6^ two risk factors that could be associated with long-term care.^7^, ^8^, ^9^ Payette et al^10^ indicated that determinants of healthy eating in community-dwelling older people can be divided into two categories: individual determinants and collective determinants. Individual determinants include determinants such as sex, age, health condition, knowledge, educational attainment, income, and living arrangement, and collective determinants include food-shopping environment, marketing of the “healthy food” message, and social support. In previous studies, individual determinants have been associated with dietary variety.^10^, ^11^ Regarding a collective determinant (food-shopping environment), Yakushiji^12^ reported that in 2010 in Japan, there were 3.82 million people aged 65 years or older who had difficulty accessing food stores. Among these, 2.02 million lived in rural areas; however, the number in urban areas is expected to increase in the future. Yakushiji also indicated that lack of food access decreased the dietary variety of the older people. Iwama^13^ has shown that older Japanese individuals in an older commuter town who had supportive relationships with neighbors had higher dietary variety than those who did not. Another Japanese study by Hanibuchi et al^14^ showed that high access to supermarkets was associated with overweight or obesity, but not with underweight. In contrast, previous studies from the United Kingdom and the United States found that low food access occurred in urban areas where the poor live — known as “food deserts” — and contributed to diet-related outcomes, such as obesity.^15^, ^16^ Thus, the effect of food access differs across nations.

The aim of the present study was to examine the association of perceived food availability and neighbor relationships on eating and underweight for the Japanese community-dwelling older people using large-scale data.

## Methods

The Japan Gerontological Evaluation Study conducted a large-scale postal survey of community-dwelling people aged 65 years or older who were not eligible to receive public long-term care benefits. The data were collected in 31 municipalities (12 prefectures) in Japan from August 2010 through January 2012. The self-administered questionnaire was mailed randomly to 169,215 subjects in 15 large municipalities and to all eligible subjects in 16 small municipalities. The municipalities were urban cities (such as Kobe and Nagoya), local cities, and rural towns/villages and were located in the prefectures of Hokkaido, Aomori, Miyagi, Yamanashi, Chiba, Aichi, Mie, Nara, Hyogo, Okayama, Nagasaki, and Okinawa. The response rate was 66.3% (112,123 respondents). The analytic sample included 102,869 subjects with valid data on sex, age, and municipality. Ethical approval was obtained from the Ethics Committee of Nihon Fukushi University in July 2010 (No. 10-05).

### Variables

Eating was evaluated using monthly frequency of food intake. The frequency of intake of fruits and vegetables was assessed with the question “How often did you eat fruits and/or vegetables over the past month?” Possible responses were “Twice a day or more,” “Once a day,” “Four to six times a week,” “Two or three times a week,” “Once a week,” “Less than once a week,” or “None.” Subjects who ate fruits and vegetables at least once a day were categorized as having frequent intake, and those who ate them less than once a day were categorized as having infrequent intake. In the case of meat and fish, the question “How often did you eat meat and/or fish over the past month?” was used, and subjects' responses were categorized in the same way as those for fruits and vegetables.

Body mass index (BMI; kg/m^2^) was calculated as weight (kg) divided by the square of height (meters) from subjects' self-reported values. Values that were not within the 4 standard deviations of the mean of those reported in the National Health and Nutrition Survey in Japan^17^ by age and sex were excluded. The analytic sample was limited to subjects whose BMI was classified as underweight (BMI <18.5) or normal weight (18.5 ≤ BMI <25).

The perceived availability of food was assessed using the question “Are stores or facilities that sell fresh fruits and vegetables present within 1 km of your home?” Possible responses were “Many,” “Some,” “Few,” “None,” or “I don't know.” Subjects who answered “Many” or “Some” were categorized as having high access, and respondents who answered “Few,” “None,” or “I don't know” were categorized as having low access. Relationships with neighbors were assessed using the question “What kind of relations do you have with people in your neighborhood?” Possible responses were “Mutual consultation, lending and borrowing daily commodities, cooperation in daily life,” “Standing and chatting frequently,” “No more than exchanging greetings,” or “None, not even greetings.” Subjects who answered “Mutual consultation, lending and borrowing daily commodities, cooperation in daily life” or “Standing and chatting frequently” were categorized as having a high level of relationship with neighbors.^18^ Subjects who answered “No more than exchanging greetings” or “None, not even greetings” were categorized as having a low level of relationship with neighbors.

Subjects were categorized by age as <75 years old or ≥75 years old, and by living arrangement as living alone or not living alone. Annual equivalent income (million yen per year) was calculated by dividing pre-tax household income by the square root of the number of household members, and the annual equivalent income was then categorized into four groups: ≥4, <4 and ≥ 2, <2, and missing data. Educational attainment was categorized as <10 years or ≥10 years. Missing data were excluded, with the exception of annual equivalent income. Because there were a large number of subjects with data missing for this metric, a “missing data” category was created.

### Statistical analysis

Proportion of low access to food stores, low level of relationship with neighbors, infrequent intake of fruit and vegetables and meat and fish, and other variables between men and women was assessed using the chi-square test. The odds ratios (ORs) with 95% confidence intervals (CIs) for infrequent intake of fruits and vegetables, infrequent intake of meat and fish, and underweight were determined using logistic regression analysis stratified by sex. The independent variables were age (reference: <75 years), food access (reference: high), relationships with neighbors (reference: high level), living arrangement (reference: not living alone), annual equivalent income (reference: ≥4 million yen per year), and educational attainment (reference: ≥10 years). Each variable was analyzed with adjustment for age in model 1, and all independent variables were analyzed simultaneously in model 2.

Furthermore, we conducted an analysis stratified by level of relationship to compare the OR of low food access between people having high levels of relationship with neighbors with those having low levels of relationship. The interaction between food access and neighbor relationships was also evaluated by including the interaction terms. Statistical significance was set at *P* < 0.05 (two-sided). All analyses were performed using SPSS Statistics 22 (IBM, Armonk, NY, USA).

## Results

The proportion of community-dwelling men and women with low food access was 25–30%. The proportion having a low level of relationship with neighbors was higher among men than among women (31% vs 19%; Table 1).

**Table 1:** Descriptive statistics of study subjects.

		n	%	n	%	*P* value
	**Men**	47,289		**Women**	55,580	
Age, years	≥75	19,813	41.9	24,639	44.3	<0.001
Access to food stores	low	11,266	24.5	15,250	28.5	<0.001
Neighbor relationships	low level	13,699	30.9	9479	18.9	<0.001
Intake of fruit and vegetables	<1/day	11,216	25.3	8636	16.5	<0.001
Intake of meat and fish	<1/day	27,532	62.4	29,856	57.4	<0.001
Body mass index	Underweight	2530	7.2	4632	11.3	<0.001
Living arrangement	Alone	3534	7.6	9618	17.7	<0.001
Annual equivalent income, million yen/year	≥4	4625	9.8	4350	7.8	<0.001
	<4, ≥2	16,535	35.0	14,950	26.9	<0.001
	<2	19,861	42.0	22,692	40.8	
	Missing	6268	13.3	13,588	24.4	
Educational attainment, years	<10	20,683	44.7	27,540	51.3	<0.001

Low food access was associated with infrequent intake of fruits and vegetables (model 2 OR 1.18; 95% CI, 1.12–1.25 for men and OR 1.26; 95% CI, 1.19–1.33 for women; Table 2) and of meat and fish (model 2 OR 1.15; 95% CI, 1.10–1.21 for men and OR 1.17; 95% CI, 1.12–1.22 for women; Table 3). However, low food access was not significantly associated with underweight (model 2 OR 1.10; 95% CI, 1.00–1.22 for men and OR 1.05; 95% CI, 0.98–1.13 for women; Table 4).

**Table 2:** Odds ratios for infrequent intake of fruits and vegetables.

		Model 1		Model 2	
		OR^a^	95% CI	OR^b^	95% CI
**Men**					
Age, years	<75	1.00		1.00	
	≥75	0.75	0.72–0.79	0.68	0.65–0.72
Access to food stores	high	1.00		1.00	
	low	1.23	1.17–1.30	1.18	1.12–1.25
Neighbor relationships	high level	1.00		1.00	
	low level	1.43	1.37–1.50	1.38	1.31–1.45
Living arrangement	Not alone	1.00		1.00	
	Alone	2.30	2.13–2.47	2.11	1.95–2.29
Annual equivalent income, million yen/year	≥4	1.00		1.00	
	<4, ≥2	1.22	1.12–1.32	1.16	1.06–1.26
	<2	1.78	1.63–1.93	1.58	1.45–1.73
	Missing	2.06	1.87–2.27	1.63	1.47–1.81
Educational attainment, years	≥10	1.00		1.00	
	<10	1.44	1.38–1.50	1.32	1.25–1.38
**Women**					
Age, years	<75	1.00		1.00	
	≥75	1.00	0.96–1.05	0.88	0.84–0.93
Access to food stores	high	1.00		1.00	
	low	1.31	1.25–1.38	1.26	1.19–1.33
Neighbor relationships	high level	1.00		1.00	
	low level	1.64	1.55–1.73	1.57	1.48–1.67
Living arrangement	Not alone	1.00		1.00	
	Alone	1.11	1.05–1.18	1.00	0.94–1.07
Annual equivalent income, million yen/year	≥4	1.00		1.00	
	<4, ≥2	1.09	0.98–1.22	1.08	0.96–1.22
	<2	2.09	1.88–2.33	1.85	1.65–2.07
	Missing	2.26	2.02–2.52	1.90	1.68–2.14
Educational attainment, years	≥10	1.00		1.00	
	<10	1.89	1.80–1.99	1.74	1.65–1.83

CI, confidence interval; OR, odds ratio.

a OR was adjusted for age.

b OR was adjusted for age, access to food stores, neighbor relationships, living arrangement, annual equivalent income, and educational attainment.

**Table 3:** Odds ratios for infrequent intake of meat and fish.

		Model 1		Model 2	
		OR^a^	95% CI	OR^b^	95% CI
**Men**					
Age, years	<75	1.00		1.00	
	≥75	0.88	0.84–0.91	0.80	0.76–0.83
Access to food stores	high	1.00		1.00	
	low	1.18	1.13–1.23	1.15	1.10–1.21
Neighbor relationships	high level	1.00		1.00	
	low level	1.02	0.98–1.06	1.01	0.96–1.05
Living arrangement	Not alone	1.00		1.00	
	Alone	1.21	1.12–1.30	1.14	1.05–1.24
Annual equivalent income, million yen/year	≥4	1.00		1.00	
	<4, ≥2	1.26	1.18–1.35	1.23	1.15–1.32
	<2	1.76	1.64–1.88	1.59	1.48–1.70
	Missing	1.80	1.66–1.96	1.57	1.44–1.72
Educational attainment, years	≥10	1.00		1.00	
	<10	1.53	1.47–1.59	1.43	1.37–1.49
**Women**					
Age, years	<75	1.00		1.00	
	≥75	1.05	1.01–1.09	0.93	0.89–0.96
Access to food stores	high	1.00		1.00	
	low	1.23	1.18–1.28	1.17	1.12–1.22
Neighbor relationships	high level	1.00		1.00	
	low level	1.15	1.10–1.21	1.11	1.06–1.17
Living arrangement	Not alone	1.00		1.00	
	Alone	1.29	1.23–1.35	1.22	1.15–1.28
Annual equivalent income, million yen/year	≥4	1.00		1.00	
	<4, ≥2	1.10	1.03–1.18	1.09	1.02–1.18
	<2	1.94	1.81–2.07	1.68	1.56–1.81
	Missing	1.94	1.81–2.09	1.66	1.53–1.79
Educational attainment, years	≥10	1.00		1.00	
	<10	2.03	1.96–2.10	1.91	1.84–1.99

CI, confidence interval; OR, odds ratio.

a OR was adjusted for age.

b OR was adjusted for age, access to food stores, neighbor relationships, living arrangement, annual equivalent income, and educational attainment.

**Table 4:** Odds ratios for underweight compared with normal weight.

		Model 1		Model 2	
		OR^a^	95% CI	OR^b^	95% CI
**Men**					
Age, years	<75	1.00		1.00	
	≥75	1.97	1.82–2.14	1.97	1.81–2.15
Access to food stores	high	1.00		1.00	
	low	1.15	1.05–1.26	1.10	1.00–1.22
Neighbor relationships	high level	1.00		1.00	
	low level	1.30	1.19–1.42	1.25	1.14–1.37
Living arrangement	Not alone	1.00		1.00	
	Alone	1.32	1.15–1.52	1.25	1.07–1.45
Annual equivalent income, million yen/year	≥4	1.00		1.00	
	<4, ≥2	1.07	0.91–1.26	1.05	0.89–1.24
	<2	1.30	1.11–1.51	1.25	1.06–1.47
	Missing	1.32	1.10–1.58	1.24	1.02–1.51
Educational attainment, years	≥10	1.00		1.00	
	<10	1.10	1.02–1.20	1.07	0.98–1.17
**Women**					
Age, years	<75	1.00		1.00	
	≥75	1.59	1.50–1.69	1.62	1.51–1.73
Access to food stores	high	1.00		1.00	
	low	1.05	0.98–1.13	1.05	0.98–1.13
Neighbor relationships	high level	1.00		1.00	
	low level	1.39	1.29–1.50	1.38	1.27–1.49
Living arrangement	Not alone	1.00		1.00	
	Alone	1.10	1.02–1.19	1.07	0.98–1.16
Annual equivalent income, million yen/year	≥4	1.00		1.00	
	<4, ≥2	1.15	1.02–1.31	1.20	1.04–1.37
	<2	1.19	1.05–1.35	1.25	1.09–1.43
	Missing	1.16	1.02–1.32	1.24	1.08–1.44
Educational attainment, years	≥10	1.00		1.00	
	<10	0.84	0.79–0.90	0.83	0.78–0.89

CI, confidence interval; OR, odds ratio.

a OR was adjusted for age.

b OR was adjusted for age, access to food stores, neighbor relationships, living arrangement, annual equivalent income, and educational attainment.

Low level of relationship with neighbors was associated with infrequent intake of fruits and vegetables (model 2 OR 1.38; 95% CI, 1.31–1.45; Table 2), but not associated with infrequent intake of meat and fish (model 2 OR 1.01; 95% CI, 0.96–1.05; Table 3) in men. In women, low level of relationship with neighbors was associated with infrequent intake of fruits and vegetables (model 2 OR 1.57; 95% CI, 1.48–1.67; Table 2), and with infrequent intake of meat and fish (model 2 OR 1.11; 95% CI, 1.06–1.17; Table 3). Low level of relationship with neighbors was also associated with underweight in both sexes (model 2 OR 1.25; 95% CI 1.14–1.37 for men and OR 1.38; 95% CI 1.27–1.49 for women; Table 4).

Living alone was strongly associated with infrequent intake of fruits and vegetables in men, but not in women (model 2 OR 2.11; 95% CI, 1.95–2.29 for men and OR 1.00; 95% CI, 0.94–1.07 for women; Table 2). Low equivalent income was associated with infrequent intake of fruits and vegetables (model 2 OR [<4, ≥2] 1.16; 95% CI, 1.06–1.26, OR [<2] 1.58; 95% CI, 1.45–1.73, and OR [Missing] 1.63; 95% CI, 1.47–1.81 for men and OR [<4, ≥2] 1.08; 95% CI, 0.96–1.22, OR [<2] 1.85; 95% CI, 1.65–2.07, and OR [Missing] 1.90; 95% CI, 1.68–2.14 for women; Table 2]. Lower educational attainment was associated with underweight in women, but not in men (model 2 OR 0.83; 95% CI, 0.78–0.89 for women and OR 1.07; 95% CI, 0.98–1.17 for men; Table 4).

ORs stratified by level of relationship with neighbors are shown in Table 5. Association between low food access and infrequent intake of fruits and vegetables was significantly higher among men with a low level of relationship with neighbors (OR 1.34; 95% CI, 1.23–1.46) than among men with a high level of relationship (OR 1.10; 95% CI, 1.03–1.18). A similar association was observed for infrequent intake of meat and fish among men (OR 1.28; 95% CI, 1.17–1.40 for people with a low level of relationship and OR 1.10, 95% CI, 1.03–1.16 for people with a high level of relationship). Among women, although similar associations were observed for infrequent intake of fruits and vegetables, the associations were not statistically significant.

**Table 5:** Odds ratios of low access to food stores stratified by neighbor relationships (dependent variables: intake of fruits and vegetables, intake of meat and fish, and underweight).

		Infrequent intake of fruits and vegetables <1/day			Infrequency intake of meat and fish <1/day			Underweight		
		OR^a^	95% CI	*P* value for interaction	OR^a^	95% CI	*P* value for interaction	OR^a^	95% CI	*P* value for interaction
**Men**										
**High level of neighbor relationships**				<0.001			0.002			0.188
Access to food stores	high	1.00			1.00			1.00		
	low	1.10	1.03–1.18		1.10	1.03–1.16		1.05	0.93–1.19	
**Low level of neighbor relationships**										
Access to food stores	high	1.00			1.00			1.00		
	low	1.34	1.23–1.46		1.28	1.17–1.40		1.19	1.02–1.40	
**Women**										
**High level of neighbor relationships**				0.056			0.203			0.619
Access to food stores	high	1.00			1.00			1.00		
	low	1.22	1.14–1.30		1.15	1.10–1.21		1.04	0.95–1.13	
**Low level of neighbor relationships**										
Access to food stores	high	1.00			1.00			1.00		
	low	1.38	1.23–1.54		1.23	1.12–1.36		1.09	0.94–1.26	

CI, confidence interval; OR, odds ratio.

a Odds ratio was adjusted for age, access to food stores, living arrangement, annual equivalent income, and educational arrangement.

## Discussion

This is the first study to use large-scale data to investigate the association of food access and neighbor relationships with diet and underweight among older Japanese. We found significant interactions of food access and neighbor relationships with diet among men.

We observed that low food access was significantly associated with infrequent eating. Although the measures used were simple — monthly intake frequency of each food category — our results are broadly consistent with those of previous studies in Japan^12^ and western settings^19^, ^20^ that used more comprehensive dietary assessments, such as the Healthy Eating Index^19^ or a total dietary variety score.^12^, ^20^

Low food access was not shown to be significantly associated with underweight. This finding is consistent with that of a previous Japanese study in limited regions,^14^ although the method of assessing food access was different. We used the perceived availability of facilities selling fresh fruits and vegetables within 1 km of the respondent's home; however, the previous work used objective measures of availability, such as the distance to the nearest supermarket and the number of supermarkets within a 500-m radius as determined using a geocoding procedure.

One important finding of the present study is that a high level of relationship with neighbors may relieve the infrequent eating caused by low food access. Iwama^13^ found that dietary variety is low among older people who do not know their neighbors' family structure, even when food access is high. Although the analytic sample in this study consisted of only 203 subjects in an older commuter town, our study supports its findings and expands its generalizability using large-scale data. We further observed that interaction of low food access and neighbor relationships with diet was statistically significant in men but not in women. This gender difference may have resulted because women generally have better cooking skills,^21^ more nutritional knowledge,^22^ search for health-related information to a greater degree,^23^ and make more health-related dietary plans^24^ than men do, so women may be less affected by food access and relationships with neighbors. Our findings might also suggest that men who have a low level of relationship with neighbors are the most vulnerable to food-access difficulty. These sex differences should be taken into account when considering measures for increasing dietary variety to prolong healthy life expectancy.

To our knowledge, neighbor relationships have not been examined independently as a variable in previous western studies of the association between diet and social contact. The questions used by Sahyoun et al^25^ assessed five sources of social contact, including “Visits with neighbors”; however, this item was combined with “Get together with family and friends” in the analysis, because few study subjects responded affirmatively to it. In another study,^20^ “neighbors” was combined with “close friend” in the study design. Our findings suggest that neighbors are an important variable in the local food environment in Japan.

In the present study, we focused on collective determinants of eating, such as food access and neighbor relationships, rather than individual determinants, such as living alone, annual equivalent income, and educational attainment. Even though ORs of individual determinants were mostly higher than collective ones, we believe that interventions for solving infrequent intake of food and underweight that are based in addressing collective determinants are more achievable than those based in addressing individual determinants. Iwama^13^ classified three approaches for solving food access problems: eating communally, food delivery (home-delivered food or meals), and improving access (providing mobile food vendors, buses for going to shops). Iwama also indicated that the support of individuals in the community is critically necessary for maintaining activities. We believe that investigating not only the needs of the individuals, but also their background, including community engagement, history, and surrounding environment, is necessary to solve food access problems in the future.

One strength of the present study is the generalizability of the results to older Japanese individuals, because the data were collected from municipalities of various sizes in 12 Japanese prefectures. The study area includes a wide range from the northern to southern part of Japan and from urban cities to rural areas. Another strength is that we specifically examined perceived availability of food access, which, among the five dimensions of food access detailed by Caspi et al^26^ (availability, accessibility, affordability, acceptability, and accommodation), has been found to be most positively associated with healthy diet.

A limitation of the present study is that we did not use an objective assessment of food availability, such as the distance to food stores. Studies using geographic information systems are becoming more common; however, such systems cannot capture non-geographic aspects of access, such as variety of products, food price, quality, and open hours for local stores. Accordingly, distance-based studies have shown a less consistent positive association with dietary outcomes.^26^, ^27^, ^28^, ^29^ Second, we focused on only fruits and vegetables and meat and fish intake as proxies of dietary variety and did not acquire data about other food groups in the present study. We consider that fruits and vegetables and meat and fish represent the minimum level of distinction necessary to assess dietary variety. The dietary variety questionnaire by Kumagai et al^2^ for older Japanese individuals consists of 10 main food groups (meat, eggs, fish and shellfish, milk, dark vegetables, soybean products, potatoes, fruits, seaweeds, and fats and oils). The present categories of fruits and vegetables and meat and fish cover four of these ten groups. It will be necessary to additionally include cereals in order to assess association with underweight, as this group is a major source of total energy intake in the Japanese diet and may be associated with socioeconomic status.^30^ Assessing dietary variety from a broader perspective should be studied in the future. In addition, height and weight were based on subjects' responses. It has been established that reported weight and height are often lower than the true values,^31^ especially for older individuals.^32^ However, older Japanese people aged less than 85 years have been shown to report their actual weight and height.^33^ Because the proportion of subjects who were 85 years or older was only 6.9% in the present study, the use of self-reported data likely did not have a large impact. Other limitations were that we did not consider other potentially relevant factors, such as car ownership, availability of public transportation, health conditions, or dietary knowledge and beliefs. Finally, cause and effect could not be determined because of the cross-sectional study design.

## Conclusion

Low food access was significantly associated with infrequent eating, but not shown to be significantly associated with underweight. Men with a low level of relationship with neighbors were the most vulnerable to difficulties in food access. Even if older people have food-access difficulty, improving neighbor relationships can serve as a countermeasure to this difficulty, which may decrease the prevalence of risk factors associated with long-term care in Japan.

## Conflicts of interest

None declared.
